# Treatment of tympanic membrane perforation using bacterial cellulose: a randomized controlled trial^☆^

**DOI:** 10.1016/j.bjorl.2016.05.002

**Published:** 2016-05-31

**Authors:** Zhengcai Lou

**Affiliations:** Department of Otorhinolaryngology, The Affiliated YiWu Hospital of Wenzhou Medical University, Zhejiang 322000, China

Dear Editor,

Herein, we present a review of the manuscript titled “Treatment of tympanic membrane perforation using bacterial cellulose: A randomized controlled trial” by Silveira et al.^1^

The work outlined in this manuscript was interesting. After review, we agree with the authors that bacterial cellulose is an excellent material for tympanic membrane (TM) regeneration and may be an effective alternative to conventional myringoplasty. However, we believe the following details of this study should be further clarified:

*The size of the perforation*: Although the authors reported a 100% closure rate using bacterial cellulose in 14 small chronic TM perforations, they calculated this rate by categorizing the size of the perforation as small or medium. However, they did not clearly describe the perforation diameter as a measurement (i.e., in millimeters or as a percentage of the TM). Previously published studies indicate that the use of Gelfoam or paper patch tympanoplasty on small chronic TM perforations is effective. Anders Niklasson et al.^2^ reported that Gelfoam plug myringoplasty was successful for 12 small chronic TM perforations, 2–4 mm in size. Another study by Park et al.^3^ reported a closure rate of 78.3% using cigarette paper on 23 chronic TM perforations, less than 5% of the TM in size. Similarly, Golz et al.^4^ reported a 78.3% closure rate using cigarette paper in 38 chronic TM perforations, less than 3 mm in size. It is also important to note that Gelfoam and cigarette paper are convenient, easy to use, and inexpensive compared to bacterial cellulose.

*The inclusion criteria are vague:* For the inclusion criteria, the authors stated, “40 patients with tympanic membrane perforations caused by otitis media were enrolled in a randomized controlled clinical study… Patients with marginal, damp or cholesteatoma perforations were excluded.” There was no indication of whether perforations with sclerotic plaques were included in this study. Sclerotic plaques are the primary factor affecting the success rate of myringoplasty. Some studies evaluating the use of tympanoplasty to treat chronic TMPs found that excision of sclerotic plaques improved the success rate.^5^, ^6^ Results from two studies utilizing fibroblast growth factor-2 (FGF-2) for the treatment of traumatic and chronic TMPs indicated that residual TM calcification was a significant risk factor for nonhealing.^7^, ^8^ Similarly, the results from a study investigating a large sample of spontaneously healing traumatic TMPs showed that preexisting sclerotic plaques were the primary cause of nonhealing.^9^

*The there was no detailed description of management of the perforated edge:* For the experimental group, the authors stated, “The perforated edges were scarified, and then a bacterial cellulose membrane was placed over the perforation laterally to the tympanic remains. The membrane was held in place by self-adhesion.” During myringoplasty, in the majority of cases, perforated edges are excised to create a fresh edge for the patch or temporal fascia graft of the chronic TMP.

To effectively convey the methodology utilized in this study, we believe that the authors will need to elaborate on all three of the above-mentioned points.

## Conflicts of interest

The author declares no conflicts of interest.

## References

[1] Silveira F.C., Pinto F.C., Caldas Neto S.D., Leal M.C., Cesário J., Aguiar J.L. (2015). Treatment of tympanic membrane perforation using bacterial cellulose: a randomized controlled trial. Braz J Otorhinolaryngol.

[2] Niklasson A., Tano K. (2011). The Gelfoam^®^ plug: an alternative treatment for small eardrum perforations. Laryngoscope.

[3] Park S.N., Kim H.M., Jin K.S., Maeng J.H., Yeo S.W., Park S.Y. (2015). Predictors for outcome of paper patch myringoplasty in patients with chronic tympanic membrane perforations. Eur Arch Otorhinolaryngol.

[4] Golz A., Goldenberg D., Netzer A., Fradis M., Westerman S.T., Westerman L.M. (2003). Paper patching for chronic tympanic membrane perforations. Otolaryngol Head Neck Surg.

[5] Migirov L., Volkov A. (2009). Influence of coexisting myringosclerosis on myringoplasty outcomes in children. J Laryngol Otol.

[6] Aslan H., Katilmiş H., Oztürkcan S., Ilknur A.E., Başoğlu S. (2010). Tympanosclerosis and our surgical results. Eur Arch Otorhinolaryngol.

[7] Hakuba N., Hato N., Okada M., Mise K., Gyo K. (2015). Preoperative factors affecting tympanic membrane regeneration therapy using an atelocollagen and basic fibroblast growth factor. JAMA Otolaryngol Head Neck Surg.

[8] Lou Z., Yang J., Tang Y., Xiao J. (2015). Risk factors affecting human traumatic tympanic membrane perforation regeneration therapy using fibroblast growth factor-2. Growth Factors.

[9] Lou Z.C., Tang Y.M., Yang J. (2011). A prospective study evaluating spontaneous healing of aetiology, size, and type-different groups of traumatic tympanic membrane perforation. Clin Otolaryngol.

